# Infant exposure to Fluvoxamine through placenta and human milk: a case series - A contribution from the ConcePTION project

**DOI:** 10.3389/fpsyt.2023.1167870

**Published:** 2023-05-18

**Authors:** Anaëlle Monfort, Evelina Cardoso, Chin B. Eap, Céline J. Fischer Fumeaux, Myriam Bickle Graz, Mathilde Morisod Harari, Etienne Weisskopf, Peggy Gandia, Karel Allegaert, Hedvig Nordeng, Jean-Michel Hascoët, Olivier Claris, Manuella Epiney, Chantal Csajka, Monia Guidi, Ema Ferreira, Alice Panchaud

**Affiliations:** ^1^CHU Sainte-Justine, Montréal, QC, Canada; ^2^Faculty of Pharmacy, Université de Montréal, Montréal, QC, Canada; ^3^Service of Pharmacy, Lausanne University Hospital and University of Lausanne, Lausanne, Switzerland; ^4^Center for Research and Innovation in Clinical Pharmaceutical Sciences, University Hospital and University of Lausanne, Lausanne, Switzerland; ^5^School of Pharmaceutical Sciences, University of Geneva, Geneva, Switzerland; ^6^Institute of Pharmaceutical Sciences of Western Switzerland, University of Geneva, University of Lausanne, Geneva, Switzerland; ^7^Unit of Pharmacogenetics and Clinical Psychopharmacology, Department of Psychiatry, Lausanne University Hospital, Lausanne, Switzerland; ^8^Clinic of Neonatology, Department Mother-Woman-Child, Lausanne University Hospital, Lausanne, Switzerland; ^9^Division of Child and Adolescent Psychiatry, Lausanne University Hospital, Lausanne, Switzerland; ^10^Laboratory of Pharmacokinetics and Toxicology, Purpan Hospital, University Hospital of Toulouse, Toulouse, France; ^11^Child and Youth Institute, KU Leuven, Leuven, Belgium; ^12^Department of Development and Regeneration, KU Leuven, Leuven, Belgium; ^13^Department of Pharmaceutical and Pharmacological Sciences, KU Leuven, Leuven, Belgium; ^14^Department of Hospital Pharmacy, Erasmus MC, Rotterdam, Netherlands; ^15^Pharmacoepidemiology and Drug Safety Research Group, Department of Pharmacy, PharmaTox Strategic Initiative, Faculty of Mathematics and Natural Sciences, University of Oslo, Oslo, Norway; ^16^Department of Child Health and Development, Norwegian Institute of Public Health, Oslo, Norway; ^17^Department of Neonatology, Maternité Régionale, Université de Lorraine, Nancy, France; ^18^Department of Neonatology, Hospices Civils de Lyon, Lyon, France; ^19^Claude Bernard University, P2S 4129, Lyon, France; ^20^Department of Women, Child and Adolescent, Geneva University Hospital, Geneva, Switzerland; ^21^Service of Clinical Pharmacology, Lausanne University Hospital and University of Lausanne, Lausanne, Switzerland; ^22^Institute of Primary Health Care (BIHAM), University of Bern, Bern, Switzerland

**Keywords:** fluvoxamine, infant exposure, human milk, cord blood, lactation, pregnancy

## Abstract

**Introduction:**

Fluvoxamine is widely used to treat depression during pregnancy and lactation. However, limited data are available on its transfer to the fetus or in human milk. This case series provides additional information on the infant exposure to fluvoxamine during pregnancy and lactation.

**Case presentation:**

Two women, aged 38 and 34 years, diagnosed with depression were treated with 50 mg fluvoxamine during pregnancy and lactation. At delivery a paired maternal and cord blood sample was collected for each woman. The first mother exclusively breastfed her child for 4 months and gave one foremilk and one hindmilk sample at 2 days and 4 weeks post-partum, whereas the second mother did not breastfeed.

**Results:**

The cord to plasma concentration ratios were 0.62 and 0.48, respectively. At 2 weeks post-partum, relative infant doses (RID) were 0.47 and 0.57% based on fluvoxamine concentrations in foremilk and hindmilk, respectively. At 4 weeks post-partum, the RIDs were 0.35 and 0.90%, respectively. The child from the first mother was born healthy and showed a normal development at the 6th, 18th and 36th month follow-ups. One of the twins from the second woman was hospitalized for hypoglycemia that was attributed to gestational diabetes and low birth weight. The second one was born healthy.

**Conclusion:**

These results suggest a minimal exposure to fluvoxamine during lactation which is in accordance with previously published data. Larger clinical and pharmacokinetic studies assessing the long-term safety of this drug during lactation and the variability of its exposure through breastmilk are warranted.

## 1. Introduction

Fluvoxamine is a selective serotonin reuptake inhibitor (SSRI) marketed for almost 40 years in Switzerland to treat depression, obsessive-compulsive disorder (OCD), social anxiety disorder or panic disorder (1, 2). It is a small (318.3 g/mol) lipophilic (logP = 2.8) molecule with a plasma protein binding around 80% and a half-life of 15.6 h. This drug is well-absorbed (94%) but subject to a significant first pass hepatic metabolism (~50%), and has no pharmacologically active metabolites (1). All these characteristics make fluvoxamine a drug that can easily cross the placenta and the blood-milk barrier.

Although fluvoxamine and other SSRIs are widely used to treat depression during pregnancy and lactation (2), data on the transfer of fluvoxamine to the fetus or in human milk are scarce (3). The ratio of fluvoxamine cord blood concentrations to maternal plasma concentrations (C/M ratio) was only determined in three women and not related to the time of the latest intake of fluvoxamine, with variable values (0.71, 0.78 and 0.08) (4–6). Similarly, only six studies measured concentrations of fluvoxamine in human milk and maternal plasma among seven women and too few samples per woman were analyzed (7–12). Half of these studies were based on a single measurement while the other half were based on a 24-hour sampling. Maternal plasma concentrations ranged from 0.02 to 0.370 mg/L and human milk concentrations ranged from 0.02 to 0.425 mg/L under daily doses ranging from 50 to 250 mg. Authors calculated a milk to plasma ratio (M/P ratio) and a relative infant dose (RID) of 0.29–1.3 and 0.5–1.6%, respectively. M/P ratios based on the Area Under the Curve (AUC) were always higher than 1. All these results indicate a low transfer of fluvoxamine into breast milk. The variation between these studies could be explained by the sampling method and difference in doses, but also by interindividual variability of the transfer of fluvoxamine to the fetus and into human milk. It is therefore necessary to better assess its transfer to the fetus and into human milk and its potential effects on breastfed infants.

We describe here two cases of women treated with fluvoxamine during pregnancy and lactation, who have been included in the SSRI milk cohort aiming to characterize the pharmacokinetics of SSRIs in pregnant and lactating women (clinical trials identification number: NCT01796132). Maternal plasma, cord blood, foremilk and hindmilk were sampled at different time points. The first woman-infant dyad was followed from delivery until 36 months postpartum to assess the effects of fluvoxamine exposure on the child development. The second dyad was followed only during the first week of postpartum.

## 2. Case presentations

### 2.1. Case 1

The first patient is a 38-year-old Caucasian woman, diagnosed with depression in 2004, weighing 69 kg 4 weeks after delivery in 2014 (minus 5 kg since late pregnancy). Her depression was treated with a daily oral dose of fluvoxamine (Floxyfral^®^) 50 mg. The treatment was continued during pregnancy. She had no other comorbidity or treatment apart from folic acid that was started before pregnancy.

She gave birth at 40 weeks of gestation to a healthy girl weighing 3550 grams (z-score 0.26) and measuring 51 cm (z-score 0.25), with Apgar scores of 9-9-10 at 1, 5 and 10 min. The infant was exclusively breastfed for 4 months and then partially breastfed until 9 months of age. After 1 month of breastfeeding, the girl weighed 4080 grams. At the 6th, 18th and 36th month follow-ups, the girl's Bayley III scores were within normal limits (cognitive, language and motor scores of 106, 94 and 100 at 36 months) and both height and weight were in the 50th percentile (13).

On the day of delivery, the mother agreed to give a blood and a cord blood sample. However, she did not take her fluvoxamine dose on this day. On day 2 and week 4 post-partum, one blood and two human milk samples were collected from the mother at approximately the same time. On each occasion, the mother collected 5 to 10 mL of milk in falcon tubes immediately prior to a feed (foremilk) and at the end of the same feed (hindmilk) according to a standardized material collection protocol.

### 2.2. Case 2

The second patient is a 34 years-old woman, also Caucasian, diagnosed with an anxiety disorder in 2000, weighing 70 kg two months before delivery. Before pregnancy, she was treated with paroxetine which was replaced by sertraline and venlafaxine. Since some adverse effects occurred with these drugs (weight gain, fluid retention and dizziness), fluvoxamine (Floxyfral^®^) 50 mg once daily orally was taken throughout pregnancy. Two months before delivery, the dose of fluvoxamine was gradually reduced to 25 mg once every 2 days. At delivery, the dose was increased to 50 mg daily again. She was treated with rapid-acting and slow-acting insulin for a gestational diabetes and folic acid was started during the 4th week of pregnancy.

The patient gave birth to male twins at 37 weeks of gestation. The first child had an Apgar score of 9-9-10 at 1, 5 and 10 min, weighed 2370 g (z-score−1.33) and measured 43 cm (z-score−2.19) at birth. He was hospitalized 1 day for recurring hypoglycemia, which was attributed to his low birthweight and/or to maternal gestational diabetes. The second baby was born healthy with an Apgar score of 9-9-9 at 1, 5 and 10 min, weighing 2090 g (z-score−2.01) and measuring 42 cm (z-score−2.61), and did not require any particular treatment. Both infants were partially breastfed for the first 3 days, then breastfeeding was stopped. No follow-ups were made for the two children because of study discontinuation.

The mother gave a blood sample and a cord blood sample on the day of delivery. No human milk sample was collected as the mother stopped breastfeeding 3 days after delivery.

## 3. Methods

All samples were analyzed using liquid chromatography coupled to electrospray mass spectrometry methods detailed elsewhere (14, 15). Lower limits of quantification were 1 and 2 ng/mL in plasma and breast milk, respectively. Methods have been fully validated according to the Food and Drug Administration (FDA) and the European Medicines Agency (EMA) guidelines (16, 17).

The C/M ratio, the M/P ratio, the absolute infant dose and the RID were calculated to assess the neonate exposure to fluvoxamine. To calculate the M/P ratio, the average concentration of fluvoxamine between foremilk and hindmilk was used. The absolute infant dose was calculated by multiplying the amount of fluvoxamine found in each human milk sample (C${\;}_{\text{milk}}^{\text{i}}$) by the average theoretical amount of milk ingested by a healthy exclusively breastfed infant (V_milk_), i.e., 150 mL/kg/day (Equation 1) (18):


(1) $$\begin{array}{l} Absolute\;infant\;dose\;={C_{milk}}^{i}\times V_{milk} \end{array}$$

Finally, the RID (%) was obtained by dividing the absolute infant dose by the maternal dose in mg/kg/day (equation 2):


(2) $$\begin{array}{l} \begin{array}{l} Relative\;infant\;dose\;(RID) \end{array}\\ \begin{array}{l} \;\;\;\;\;\;=\frac{Absolute\;infant\;dose\;(mg/kg/day)\;}{Maternal\;dose\;(mg/kg/day)}\;X\;100\;\; \end{array} \end{array}$$

## 4. Results

Table 1 presents concentrations of fluvoxamine in cord blood and maternal plasma at delivery and Table 2 those in maternal plasma and human milk at week 1 and 4 postpartum, when available. In both cases, fluvoxamine concentrations were lower in cord blood than in maternal plasma with C/M ratios of 0.62 in case 1 and 0.48 in case 2, respectively. Maternal plasma concentrations gradually decreased between 10 h (56.7 ng/mL), 13 h (30.1 ng/mL) and 39 hours (23.1) ng/mL after the last dose. In human milk, concentrations measured in case 1 were higher in hindmilk than in foremilk at both week 1 and 4 post-partum, and higher in maternal plasma than in human milk with M/P ratios of 0.53 and 0.83 at each sampling time, respectively. According to equation 1, an exclusively breastfed infant who takes 150 mL/kg of milk every day would ingest between 0.0025 and 0.0065 mg/kg/day of fluvoxamine, implying that the RID would range between 0.35 and 0.90% of the weight-adjusted maternal dose.

**Table 1:** Fluvoxamine concentrations in cord blood (C) and maternal plasma (M) at delivery and corresponding C/M ratios.

**Case**	**Fluvoxamine dose (mg)**	**Type of sample**	**Time after dose (h)**	**Concentration (ng/mL)**	**C/M ratio**
Case 1	50	Cord blood	37.5	14.30	0.62
	Maternal blood	39.0	23.06		
Case 2	50	Cord blood	14.5	6.36	0.48
	Maternal blood	12.4	13.15		

**Table 2:** Fluvoxamine concentrations in maternal blood and human milk from case 1 after 1 and 4 weeks postpartum, milk to plasma (M/P) ratios and relative infant doses (RID).

**Time of sampling**	**Type of sample**	**Time after dose (h)**	**Concentration (ng/mL)**	**Absolute infant dose (mg/kg/day)**	**M/P ratio**	**RID (%)**
First week post-partum	Maternal blood	13.3	30.1		0.83	
	Foremilk	11.5	23.0	0.0035		0.47
	Hindmilk	13	27.0	0.0041		0.57
Fourth week post-partum	Maternal blood	9.7	56.7		0.53	
	Foremilk	9.9	16.9	0.0025		0.35
	Hindmilk	10.3	43.3	0.0065		0.90

## 5. Discussion

This paper presents the transfer of fluvoxamine to the fetus and in human milk based on concentrations of the drug in cord blood, maternal blood and human milk samples obtained from two women.

In our two cases, we measured C/M ratios of 0.48 and 0.62 respectively, very close to the previously reported mean C/M ratio of 0.52 (4–6). As mentioned previously, fluvoxamine has physicochemical characteristics that favorize its passive diffusion across the placenta. Even though placenta has metabolizing capacity, it is unlikely to metabolize fluvoxamine because the CYP450s responsible of fluvoxamine metabolism ate not known to have an activity in the placenta (19). Therefore, our observations are in accordance with the mechanism of drug transfer across the placenta. Although it appears to be a significant transfer of fluvoxamine in cord blood, no symptoms of withdrawal or sedation were observed in the three in utero exposed neonates. The causality between in utero exposure to fluvoxamine and the occurrence of hypoglycemia seen in one of the newborns is considered low as the mother had gestational diabetes, a well described cause for neonatal hypoglycemia, and the infant was born with a low birth weight (20). In light of available medical literature, tapering or stopping SSRI treatment a few weeks before delivery do not improve neonatal outcomes and exposes the mother to a disease decompensation during the fragile period of late pregnancy and postpartum (21).

Milk excretion of a drug in breast milk can be quantified in various ways, one of it being the M/P ratio (18). In our cases, M/P ratios were 0.53 and 0.83, indicating that fluvoxamine concentrations in plasma were higher than in human milk. Such results are in accordance with the M/P ratios obtained by previous studies (0.29 and 1.03) that have been determined based on single measurements (7, 8, 11). On the contrary, they are lower than those obtained by other studies (1.21–1.34) and based on the time-independent parameter AUC, which is an alternative method for M/P estimation (9, 10, 12). One reason for this discrepancy between studies is the difference in sampling methods, i.e. the timing of sampling. For example, our M/P ratio calculated 9.7 h after the last dose is close to the one obtained by Weissman, 9 h after the last dose (0.83 vs 1.03). In fact, milk and plasma concentrations do not increase and decrease completely in parallel, thus a M/P ratio < 1 at one point does not mean that milk concentrations are always lower than plasma concentrations over the entire dosing interval (18). Our results are compared to previous published studies in Table 3.

**Table 3:** Comparison of published studies and present work on fluvoxamine levels in maternal plasma and human milk.

**Study**	**Number of cases**	**Number of milk sample**	**Dose (mg/day)**	**Time after dose (hours)**	**Maternal blood level (mg/L)**	**Human milk level (mg/L)**	**Absolute infant dose (mg/kg/day)**	**M/P ratio**	**RID (%)**	**Adverse effects**
Wright et al. (7)	1	1	200	4.75	0.31	0.09	0.010	0.29	0.5	No
Yoshida et al. (8)	1	1	100	3.0	0.17	0.05	0.008	0.29	0.5	No
Arnold et al. (9)	1	6	75	3.75 to 23.75	0.02	0.03-0.04	0.006	1.20	0.6	No
Hägg et al. (10)	1	13	200	0.0 to 12.0	0.19-0.26	0.22-0.40	0.048	1.32	1.6	No
Weissman et al. (11)	1	1	250	9.0	0.37	0.38	0.057	1.03	1.6^**^	No
Kristensen et al. (12)	2	18	50 and 150	0.0 to 24.0	0.05-0.27^*^	0.07-0.43^*^	0.005-0.038	1.21-1.34	0.8-1.4	No
Present work	1	4	50	9.9 to 13.0	0.03-0.06	0.02-0.04	0.003-0.007	0.53-0.83	0.4-0.9	No

* Maximum concentrations of fluvoxamine in maternal blood or human milk.

** Assuming a maternal weight of 70 kg.

The exposure of newborns to a drug during breastfeeding is estimated using the RID, i.e., the percentage of the weighted maternal dose ingested by the newborn. The WHO working group suggested that if a drug has an RID of <10%, it is unlikely that an infant exposed to this drug through human milk would experience any adverse effects. Based on a daily milk intake of 150 mL/kg, we calculated that a healthy newborn would ingest between 0.35 and 0.90 % of a maternal dose of 50 mg/day. This value is far from the 10% threshold defined by the WHO, suggesting a low risk of adverse effects in breastfed infants. Furthermore, these values agree with previously published values that are between 0.5 and 1.6% (7–12). However, the RID presents multiple shortcomings and should therefore not be the only parameter considered to decide if a drug can or cannot be used during breastfeeding, especially for antidepressants. Indeed, the RID, expressed as a percentage, will not change if the maternal dose increases as the human milk concentrations will also increase. Therefore, it does not consider the drug safety in the newborn. Moreover, this 10% threshold is the same for all drugs and does not take into account the inherent toxicity, the therapeutic range, and the pharmacokinetic properties (e.g., bioavailability, half-life) of the drug in the newborn (18). Thus, all these factors and a close monitoring of the infant should be included in the assessment of the drug safety during breastfeeding.

In our study, we also observed a variation in fluvoxamine concentrations between foremilk and hindmilk. Even though the difference is minimal in the first week, the concentration is multiplied by at least 2.5 in the fourth week, following the level of fat concentration in human milk at these different time points (22). The lipophilicity and weak alkalinity of fluvoxamine can explain the increase of concentration in hindmilk as compared to foremilk (1). The difference in concentration between the 1 day and the fourth week post-partum can be explained by the milk produced, i.e., a mixture of colostrum and transitional milk that is composed of more proteins and less fat than mature milk (especially hindmilk), for which a lipophilic drug such as fluvoxamine has less affinity. At this stage, the composition of foremilk and hindmilk are more similar justifying a low increase in fluvoxamine concentrations between these two types of milk. Moreover, it is possible that tight junctions between the alveolar cells are not completely closed allowing the passage of all types of molecules regardless of their physicochemical properties and the milk composition (23, 24). Overall, the increase in fluvoxamine concentration between foremilk and hindmilk is not of concern as the RID is very low. A breastfed child will be exposed to a mixture of both and, thus, to a mean concentration of fluvoxamine between foremilk and hindmilk. Therefore, although this difference is of scientific interest, there is no reason to favor foremilk or hindmilk in the clinical setting.

The infant exposed to fluvoxamine during breastfeeding (case 1) showed no adverse effects and had a normal neurodevelopment at the 6-, 18- and 36-month follow-ups, which is consistent with previous published case reports. The twins (case 2) were not exposed to the drug through breastfeeding, and moreover the mother did not consent to developmental follow-up. The absence of observed adverse effects in the neonate (case 1) together with a calculated RID of <1% are nevertheless reassuring regarding the use of fluvoxamine during breastfeeding. Since breastfeeding has many benefits for the developing child including protection against various infections and probably a favorable effect on neurodevelopment and psychoaffective development, the risk benefit ratio is in favor of continuing breastfeeding while taking fluvoxamine (25).

Although new information was added on the transfer of fluvoxamine to the fetus and into human milk, there are some limitations to our study. First of all, C/M and M/P ratios were calculated using a limited number of samples and there is no evidence that these ratios would be similar at all timepoints over the dosing interval. To ensure a more representative assessment of these parameters, the area under the curve of fluvoxamine concentrations based on multiple timepoints or a population approach should be used, if feasible. Moreover, as our results are only based on one case and limited samples, intra and inter-individual variabilities linked to risk factors could not be determined (e.g., pharmacogenetic polymorphisms affecting the metabolism and transport of drugs and/or the response to treatment/side effects). Indeed, it was previously demonstrated that the CYP2D6 genotype significantly influence fluvoxamine concentrations, especially at a low dose of 50 mg/day, but no genotype determination was made for these two cases (26). Also, the data on infant adverse effects are limited as they are based on the one mother's impression at the follow-ups. Finally, in our study we did not analyze nursing infants' blood samples that would be the best approach to assess the infant exposure to a drug during breastfeeding.

## 6. Conclusion

In conclusion, as in previous case reports, our findings show a limited transfer of fluvoxamine into human milk with a RID below 1% and no adverse effects were observed in the one breastfed infant. These observations add to the limited available information indicating that maternal fluvoxamine treatment produces low levels in human milk and would thus not be expected to cause any adverse effects in breastfed infants. However, these results were obtained from a single mother-infant pair and limited samples. More complete pharmacokinetic studies or population pharmacokinetic studies should be performed on the transfer of fluvoxamine into cord blood and human milk to confirm our results and the safety of fluvoxamine use during pregnancy and lactation.

## Data availability statement

The original contributions presented in the study are included in the article/supplementary material, further inquiries can be directed to the corresponding author.

## Ethics statement

The studies involving human participants were reviewed and approved by the Cantonal Ethics Committee Vaud (CER-VD) (470/11). The patients/participants provided their written informed consent to participate in this study. Written informed consent was obtained from the participants for the publication of any potentially identifiable images or data included in this article.

## Author contributions

CE, CC, and AP contributed to conception and design of the study. CF, MG, MM, EW, J-MH, OC, and ME collected and analyzed the samples. AM wrote the first draft of the manuscript. All authors contributed to the analysis and the interpretation of the data, revision, read, and approved the submitted version.
